# Development and usability testing of a patient decision aid for newly diagnosed relapsing multiple sclerosis patients

**DOI:** 10.1186/s12883-019-1382-7

**Published:** 2019-07-20

**Authors:** Nick Bansback, Judy A. Chiu, Robert Carruthers, Rebecca Metcalfe, Emmanuelle Lapointe, Alice Schabas, Marilyn Lenzen, Larry D. Lynd, Anthony Traboulsee

**Affiliations:** 1 School of Population and Public Health, University of British Columbia, 2206 East Mall, Vancouver, British Columbia V6T 1Z3 Canada; 2 Centre for Health Evaluation & Outcome Sciences, St. Paul’s Hospital, 1081 Burrard Street, Vancouver, British Columbia V6Z 1Y6 Canada; 3 Division of Neurology, University of British Columbia, Djavad Mowafaghian Center for Brain Health, 2215 Wesbrook Mall, Vancouver, British Columbia V6T 1Z3 Canada; 4 Faculty of Pharmaceutical Sciences, University of British Columbia, 2405 Wesbrook Mall, Vancouver, British Columbia V6T 1Z3 Canada; 5 Collaboration for Outcomes Research and Evaluation, 2405 Wesbrook Mall, Vancouver, British Columbia V6T 1Z3 Canada; 6 Vancouver, British Columbia Canada

**Keywords:** Multiple sclerosis, Decision aid, Disease-modifying therapy, Decision making

## Abstract

**Background:**

Multiple sclerosis (MS) patients often struggle with treatment decisions, in part due to the increasing number of approved disease modifying therapies, each with different characteristics, and also since physicians can struggle to identify which of these characteristics matter most to each individual patient. Decision uncertainty can contribute to late treatment initiation and treatment non-adherence—causes of ‘undertreatment’ in MS. An interactive online patient decision aid that informs patients of their options, considers their individual preferences and goals, and facilitates conversations with their physicians, could improve how patients with relapsing forms of MS make evidence-based treatment decisions.

**Objective:**

To develop and evaluate a prototype patient decision aid (PtDA) for first-line disease modifying therapies for relapsing-remitting multiple sclerosis.

**Methods:**

Informed by previous studies and International Patient Decision Aid Standards guidelines, a prototype PtDA was developed for patients with relapsing multiple sclerosis considering first line treatment. Patients with relapsing multiple sclerosis were recruited from the University of British Columbia’s Multiple Sclerosis Clinic to participate in either an online survey or a focus group. Online survey participants completed the PtDA, followed by measures of acceptability, usability, and preparedness for decision-making, and provided general feedback. Focus group participants assessed usability of the revised PtDA. The analysis of qualitative and quantitative data led to improvements of the PtDA prototype.

**Results:**

The prototype PtDA received high ratings for acceptability and usability, and after its use, participants reported high-levels of preparedness for decision-making. Analysis of all qualitative data identified three key themes: the need for credible information; the usefulness of the PtDA; and the importance of normalizing and sharing experiences. Nine content areas were identified for revision. Overall, participants found the PtDA to be a valuable tool for facilitating treatment decisions.

**Conclusions:**

This mixed methods study has led to the development of a PtDA that can support patients with RRMS as they make treatment decisions. Future studies will assess the feasibility of implementation and the impact of the PtDA on both the timely treatment initiation and longer-term adherence.

**Electronic supplementary material:**

The online version of this article (10.1186/s12883-019-1382-7) contains supplementary material, which is available to authorized users.

## Background

Multiple sclerosis (MS) is a leading cause of non-traumatic neurological disability for young adults [1–3]. Approximately 80–85% of MS patients are diagnosed with relapsing-remitting multiple sclerosis (RRMS) and experience clearly defined disease exacerbations that last from a few hours to months [4, 5]. A window of opportunity exists in early RRMS to gain maximal benefit from disease modifying therapies (DMTs) [6, 7]. However, patients often struggle with treatment decisions and delay initiating treatment [6, 8–10]. The complexity of treatment decisions is driven by the lack of useful markers to predict the disease course in each individual patient [11] and an increasing number of DMT options, each with unique benefit and side-effect profiles including rare but potentially severe side-effects [12–14]. Further complicating the decision, routes of administration, monitoring, cost, availability, and future sequencing considerations vary across DMT options [12–14]. Both late treatment initiation and treatment non-adherence are causes of ‘undertreatment’, a persistent issue for patients with MS [8, 15].

Physicians often struggle to accurately assess patient preferences [16] and may rely on their own preferences and cognitive distortions (e.g. overconfidence, tolerance to risk and ambiguity) to make treatment recommendations [17]. These can often be incongruent with individual patient preferences [18–20] and may lead to patients being uncertain that the prescribed treatment is the best option for them [21–24]. In MS, five preference subgroups have been identified. These subgroups are characterized by varying degrees of desire to avoid risk of serious adverse events and improve symptoms, and preferences for specific routes of administration [25]. Given the diversity of preferences, understanding patient preferences may be important to increasing the number of patients that initiate and adhere to therapy long-term [26, 27]. In other chronic diseases, substantial evidence supports the use of patient decision aids (PtDAs) to promote shared decision-making between the patient and their doctors [28]. Shared decision making using PtDAs can help patients feel better informed and have a better understanding of their own values, and may improve assessment of treatment risk and quality of treatment decisions [28].

The objectives of this study were to: (1) develop a PtDA for newly diagnosed RRMS patients considering a DMT to manage their condition, and (2) investigate the usability and acceptability of the PtDA in the clinical decision-making process. Although the Cochrane review by Stacey et al. evaluated one German study on the use of an evidence-based patient decision aid on multiple sclerosis immunotherapy [22], various decision support tools have been developed to help patients make treatment decisions in MS [29–36]. However, the decision aid tools tend to focus on only a limited part of the decision (e.g. whether or not to take medication [34]), fail to ask what aspects of treatment matter most to patients, and do not help match these preferences with available DMTs [33–36]. Both initiating medication and selecting a medication are significant decisions for patients with RRMS and information alone often does not lead to appropriate decision-making [37]. To address this gap, we developed a novel online patient decision aid (RRMS-PtDA) for patients with RRMS who are considering a first-line DMT. Six first-line DMTs available to MS patients in British Columbia, Canada in March 2017 were explored, including: Avonex, Betaseron, Rebif, Copaxone, Aubagio, and Tecfidera. The beta-interferons and Copaxone are modestly effective at preventing relapses and future disability, with only marginal differences in efficacy, tolerability, and short and long-term safety [38]. The oral medications (Aubagio and Tecfidera) may have improved efficacy but also have known and unknown safety concerns. For instance, using Tecfidera carries a risk of developing progressive multifocal leukoencephalopathy (PML) [39]. Information about other treatment options from the MS Society of Canada (e.g. rehabilitation and physical therapy, relapse management therapies, symptom management therapies) were also included. A unique feature of the RRMS-PtDA is its ability to tailor the information provided to match individual patient goals and preferences and to personalize how treatment options are presented.

## Methods

### Overview

The International Patient Decision Aid Standards (IPDAS) process for systematic development of PtDAs was used to guide the development of this tool [40, 41]. Following a scoping review and assessment of the need for a PtDA for RRMS, a multidisciplinary steering committee was formed. The committee consisted of physician specialists in MS, patients, software developers, and researchers with expertise in decision-making. Ethics was obtained from the Behavioural Research Ethics Board at the University of British Columbia.

### Identification of patient preferences and physician perceptions

A previous study used qualitative individual and group interviews to identify the 6 most important aspects of treatment for patients with RRMS: (1) slowing progression of MS; (2) reducing symptoms associated with MS; (3) preventing relapse and MRI changes; (4) minimizing minor side effects; (5) avoiding serious adverse events; and (6) choosing route of administration [25]. The relative importance of each attribute was determined using a stated preference technique known as a discrete choice experiment.

To elicit clinicians’ views on patient decision support needs, a systematic literature review and individual semi-structured interviews with clinicians were conducted. In addition, direct observation of patient-clinician interactions informed development of the PtDA.

### Literature review

In collaboration with MS clinicians, a comprehensive literature search for comparative evidence was conducted to provide consistent levels of evidence to present in the PtDA. Of the available evidence, we chose to utilize findings from a review by the Canadian Agency for Drugs and Technologies in Health (CADTH) Therapeutic Review for MS [42]—a comprehensive review of the existing literature, published studies, materials and other information and documentation available to CADTH on treatments for MS. The review included a network meta-analysis that provides indirect estimates on the relative effectiveness of DMTs even where no direct trials exist. Conducted without industry funding and reviewed by health economists, methodologists, and clinical experts in MS, the CADTH report is rigorously peer reviewed, and was identified as providing the most comprehensive, accurate and unbiased evidence. Information from the MS Society of Canada [43] was used to supplement the report as needed.

### Assessment of logistical needs for clinical implementation

Qualitative feedback from patients and physicians, a literature review, a review of clinic operations, and best practice recommendations indicated that a PtDA needed to be accessible from multiple locations (e.g., home, or the physician’s office) and easy to update as new treatments and evidence become available. It was also determined that a summary sheet would be needed to provide the results of patients’ preferences and knowledge assessments to physicians prior to patient consultations. Consequently, we developed an online, interactive decision aid using a previously developed open source platform [44].

### Initial prototype development

Development of the prototype PtDA was guided by the IPDAS criteria for creating quality PtDAs [40, 41] and supported by the multidisciplinary steering group. Researcher experience with previous PtDAs also informed prototype development.

The initial PtDA was comprised of 5 sections: (1) a history module that collected a brief medical history to provide personalized information on subsequent pages; (2) an information module that presented effectiveness and side effect profiles of the 6 first-line DMTs available to patients in British Columbia, Canada in March 2017; (3) an interactive value clarification module that guided patients to consider which attributes of treatment matter most to them; (4) a decision module that compared the treatment options and suggested a treatment that best fit the patient based on information provided in modules 1 and 2; and (5) a tailored summary screen that described patient health status, preferred treatment choice, and any questions or concerns the patient had for their health care team—the summary could be printed or e-mailed directly to the MS clinic to be included in patient electronic medical records. Videos of a physician explaining the importance and goal of each section were also included as an alternative to reading text only.

The prototype was then reviewed by a team of physicians specializing in the diagnosis and treatment of MS (RC, EM and AT). Physician feedback focused on two areas: evidence on drug effectiveness and side effects; and development of appropriate questions about patient medical history. Several changes to the prototype were made to address the physician feedback.

### Quantitative usability testing

A web-survey assessed the acceptability, usability, and preliminary usefulness of the prototype PtDA. Faulker et al. [45] suggest using a sample of 10–20 participants in usability studies to identify 80–95% of usability problems. We recruited 18 patients from the University of British Columbia Multiple Sclerosis Clinic (UBC MS Clinic) who were eligible to participate if they: were at least 18 years of age; had a diagnosis of RRMS; could read, speak and understand English, use a computer interface, and provide informed consent; and were treatment naïve or had not used treatment for at least 2 years. This group of patients has been identified to obtain a diverse range of perspectives to identify usability issues. Patients with other forms of MS, such as clinically isolated syndrome and inactive progressive disease were not eligible for this study due to treatment eligibility criteria from local health authorities. Patients meeting inclusion criteria were invited to participate in the study by the research assistant. Those who consented to participating received an email invitation with the link to the survey and decision aid to complete. Participants completed the PtDA followed by the scales below, and open-ended questions on clarity, usability, and perceived need for the PtDA:

#### System usability scale (SUS)

Developed in 1986, the 10-item SUS uses a 5-point Likert scale (anchored at “Strongly Disagree” and “Strongly Agree”) to assess subjective usability of a product. Scores range from 40 to 100; a score below 68 indicates below average usability, and a score at or above 68 indicates above average usability [46, 47].

#### Acceptability scale

Developed specifically for PtDAs, the acceptability scale assesses how well information is presented for different topics (e.g. risk factors, treatment etc.) and overall impressions of the decision tool. There is no total score for the Acceptability Scale. Instead, items are analyzed individually [48]. The Acceptability Scale is designed to accommodate any health decision and has been used in many different contexts including atrial fibrillation [49]; prenatal testing [50]; and lung cancer [51].

#### Preparation for decision making scale (PDMS)

The PDMS assesses patient perception of the usefulness of a PtDA in preparing them to consult with physicians and make a healthcare decision. The 10-items of the PDMS are rated for agreement using a 5-point Likert scale (“Not at all”; “A little”; “Somewhat”; “Quite a bit”; and “A great deal”) [52]. Items are summed to give a score ranging from 0 to 100, with higher scores indicating greater perceived preparedness for decision-making. The PDMS is well validated, with high discriminant validity and both internal and test-retest reliability [53, 54].

### Qualitative usability testing

A focus group was conducted to further assess usability and to gain insight on the feedback and findings from the survey. Patients were recruited from the UBC MS Clinic and were eligible to participate if they: had ever received a diagnosis of RRMS; could use a computer interface, read, speak, and understand English, and provide informed consent; and were at least 18 years of age. As the purpose of the focus group was to assess usability, current treatment was not an exclusion criterion. In total, 7 patients participated in the focus group. After providing consent, participants were e-mailed a link to the PtDA one week before the focus group and instructed to complete the PtDA before attending. All participants completed a brief demographic survey. The focus group was 90-min in duration and was led by a facilitator that was not involved in the development of the tool.

### Analysis

Descriptive statistics were used to assess participant characteristics as well as scores from the SUS, Acceptability Scale, and PDMS. Where possible, mean scores were compared to the standards identified in the literature (i.e., SUS [46, 47]). All scale scores were interpreted per the scale user manual.

Discussions from the focus group were transcribed and analyzed. Focus group transcripts and the open-ended questions from the survey were analyzed using conventional content analysis [55]. Transcripts were analyzed using inductive coding, conducted in an iterative fashion. Constant comparison was used to maximize parsimony and coherence. Codes were then reviewed by an independent researcher who did not participate in the survey or the focus group. Any coding concerns were resolved by discussion. Lastly, as recommended by Lincoln and Guba [56], member-checking was used to ensure that results were credible and confirmable.

## Results

### Participant characteristics

In total, 25 individuals with MS participated (*n* = 18 for the survey; *n* = 7 for focus groups). Participants ranged from 21 to 70 years of age and reported disease durations of less than 1 year to more than 17 years, indicating a broad representation of disease progression and time since diagnosis. Patient determined disease steps from MS ranged from no limitation to significant limitation requiring bilateral walking support for short distances [57, 58]. As in other cohorts of MS patients [59], the majority of participants were female.

### Quantitative prototype testing

#### Usability

The SUS yielded a mean score of 80.6 (*s* = 13.7), indicating that the tool had above average usability (Table 1). Analysis of individual items found that 94% of participants rated all items 3 or better on a 5-point scale, indicating consistent usability across features of the PtDA. Importantly, 100% of participants rated the PtDA “easy to use”, “well integrated”, and consistent (Additional file 1: Table S1).

**Table 1:** Summary of results from the system usability, acceptability, and preparation for decision-making scales used

Scale	N = 18
System Usability Scale	
Mean (SD)	80.6 (13.7)
Above average usability, n (%)	17 (94%)
Acceptability Scale	
Amount of information: *About right*, n (%)	13 (72%)
Balanced presentation of information: *Completely balanced*, n (%)	12 (67%)
PtDA fits patients’ discussions with the physician, nurse, or pharmacist: *As it is or with some alteration*, n (%)	16 (89%)
Icons were readable, n (%)	18 (100%)
Words in the PtDA made sense, n (%)	18 (100%)
Willing to use PtDA/tell someone about it, n (%)	18 (100%)
Preparation for Decision Making Scale	
Mean (SD)	76.8 (15.3)
Range (0–100)	50 to 100

*SD* Standard Deviation, *PtDA* Patient Decision Aid

#### Acceptability

All items of the Acceptability Scale were rated positively. The majority of patients reported that the PtDA presented the right amount of information (72%; *n* = 13), with a subset of patients reporting that the PtDA presented slightly less information than desired (22%; *n* = 4). Likewise, most patients reported that information was presented in a balanced manner (67%; *n* = 12); however, one third reported that the PtDA was slightly or clearly biased towards taking treatment (*n* = 6). Half of patients reported that the information presented aligned with the information they had received from other health professionals (*n* = 9), however 39% of patients (*n* = 7) reported some discrepancies between the PtDA and information received from other health professionals, and 11% (*n* = 2) reported no alignment between the two sources of information (Table 1, Additional file 1: Table S2). Patients rated the clarity of the information highly; 75% of content areas were rated as having “fair” clarity or above by all respondents. Two items—information on funding and authors, and references—were each rated “poor” clarity by 1 participant (6%) (Additional file 1: Table S3).

#### Preparation for decision-making

Analysis of PDMS scores found a mean PDMS rating of 76.3 (*s* = 15.3), indicating a high level of perceived preparedness to make a treatment decision after completing the PtDA (Table 1). See Additional file 1: Table S4 for participant ratings of individual items.

### Qualitative assessment of the PtDA

Content analysis of the focus group transcript and open-ended text responses identified three overarching themes: (1) need for information; (2) usefulness of the PtDA; and (3) importance of normalizing and sharing experiences.

#### Theme 1: need for information that the PtDA provides

Focus group participants who had previously made treatment decisions before the PtDA was available reported that the physician generally provided information orally. Upon receiving a diagnosis and learning of treatment options, participants were not provided with information booklets or other informational materials that could be taken home. As a result, several participants reported that early appointments were difficult: the quantity of information was overwhelming; and it was a challenge to keep track of the questions they had asked or wanted to ask. These participants also reported trying to find information online but found that it was hard to find trustworthy information. Participants who were more recently diagnosed reported difficulty compiling and comparing the information they found in a meaningful way. Feedback and discussion emphasized that participants wanted credible information that they could reflect on after their initial appointment with a neurologist.

*“I still think like with my neurologist, it [getting information] was largely in person…my girlfriend was trying to keep up with her [the neurologist] and I think I probably remember one sentence that she said so it was all kind of overwhelming.”*

*“Well the problem was that at that time too, what you would see online, you couldn’t believe a lot of it because there was a lot of stuff [like], “Send us this money and we’ll cure you” sort of thing where that I don’t think quite exists like it used to.”*

#### Theme 2: usefulness of the decision aid

The feedback on the usefulness of the PtDA was overwhelmingly positive. Focus group and open-ended responses suggested that the tool filled a need; it provided credible information, in an accessible manner, at a time when patients are feeling overwhelmed. Participants reflected on the impact that this tool would have had or did have on their decision-making process.

*“The decision aid cut my research in half.”*

*“So, at our first meeting, we probably spent way too much time talking about the different treatments. I didn’t have anything like this, this little table. That would have been like a godsend.”*

#### Theme 3: importance of normalizing and sharing experiences

Focus group participants also reported that the PtDA may provide a unique opportunity to normalize and share experiences with those newly diagnosed with MS. Participants suggested replacing existing videos of the physician describing the importance of each section with patients sharing their experiences of living with the disease and strategies they tried. This opportunity was particularly important because participants reported having limited contact with people with lived experience of MS, and instead were often inundated with suggestions from family and friends. Importantly, for focus group participants this normalization was not about the disease symptoms but rather about the emotional challenges that can arise when receiving a diagnosis.

*“[It would be helpful to say] this decision doesn’t have to be made today. It is okay to take some time.”*

*“And I think that, you know, you sort of touched on this, that overwhelming. It’s normal to feel overwhelmed with all this information.”*

### Modifications

**Fig. 1:**
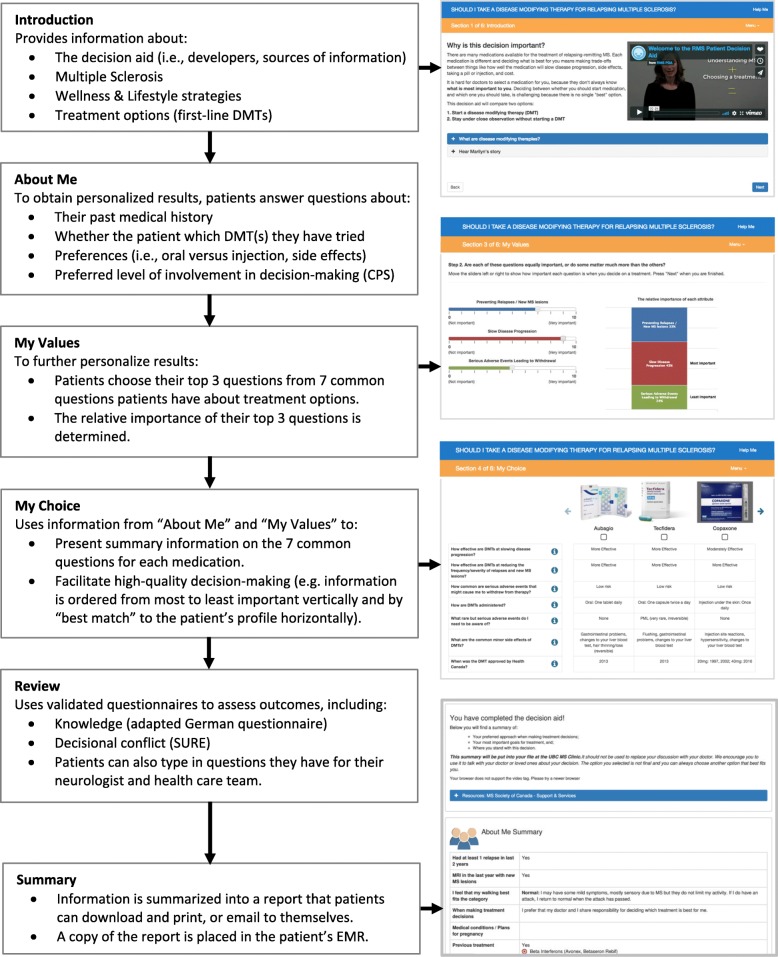
Outline of the Relapsing Multiple Sclerosis Patient Decision Aid

Based on the feedback from the quantitative and qualitative work, eight content areas were identified for improvement. These included: clarity; content; content presentation; video content; hyperlinks; functionality; value elicitation method; and summary. For most content areas, participant feedback was largely consistent and specific—for example, 5 out of 7 focus group patients suggested the ability to download a 1-page reference chart comparing all medications. Illustrative quotations, and changes made to address feedback are listed in Table 2 and screenshots from the revised PtDA are shown in Fig. 1.

**Table 2:** Usability issues from prototype testing and focus group participants and recommended revisions

Area	Usability Issue	Recommended changes
Clarity	“Informative and very technical. Make it easier to understand – patient friendly.”	*Simplify language.*
	“… [M]ore consistent separation between steps to inform versus decision making.”	*Limit the amount of information on each page and present one question per page.*
	“[A]fter selecting from this page “ABOUT ME”, I thought “OPTIONS” were the selection of medications made for me. Especially because it personalizes the top of the page then continues on with Medications, so I assumed these were choices made for me. Eventually, of course, I realized “MY CHOICE” was the medication choices that were recommended for me.”	*Revise ordering of information. Show information about treatment options in the “Introduction”, ask “About Me” questions, and then show best matches in “My Choice”.*
	“I found it confusing [referring to ‘doctor recommended’ treatment label]. I was like, what is best match over doctor recommended?”	*Remove doctor recommended label for treatment options.*
Content	“I’m not sure if I could clearly show what my symptoms have been since I was diagnosed.”	*Add an open-ended question that allows patients to share their most concerning symptoms with the doctor.*
	“I would have liked to have more options to choose from for some of the questions.”	*Add more response options to questions.*
	“It is important to me to know how long a drug had been on the market. I wanted to know the safety of the drug had been tested over a long period of time and that was part of my decision process.”	*Add Health Canada approval date for each medication.*
	“And this is something that I’ve only ever seen talked about in one forum, like one of those physician forums. What is the actual impact of the treatment on the quality of life?”	*Where possible, add information on the impact of treatment on quality of life to presentation of treatment options.*
	“I feel like being quizzed [all participants agree] on how well you read everything and memorized it. “I think if you’re developing cognitive challenges, that would be a bit complicated.”	*Revise quiz to be less burdensome.*
Content Presentation	“Maybe have a grid of all medications laying out all relative details so that they can all be compared side by side instead of using the boxes that dropped down to read about them.” (5 patients)	*Create a 1-page comparison chart that patients can access and save for their own use.*
	“[A] more detailed summary of the treatment options selected at the end.” “Being able to consolidate the information with my doctor or other healthcare professionals would allow for optimal understanding and learning”	*Improve summary page to contain detail patients want to see, and questions they might have for their doctor.*
	“I have to admit, the colour is an issue. …It definitely was a bit of a challenge with the bars and the colour contrasts…For me, if there’s not a strong enough contrast, it’s quite difficult to see.”	*Improve contrast in information presentation. Darken background colour for information headings.*
Video Content	“I didn’t think it [the video] added anything to it to it. It was fine, but I don’t know if it was necessary.”	*Remove video describing MS.*
	“Well it [personal accounts] seems like a real thing as opposed to just the information. Like someone in your situation actually is saying that.” “I think a testimonial would be a lot different than a factual video. I think it would be helpful to hear… And how it affected them, and how they felt about it.”	*Include patient accounts of receiving an MS diagnosis, and the experience of choosing a treatment.*
Links	“Good content. I liked the links to sources and studies, and found myself clicking to those links to find out more info. I would have liked to see a few more. Example, when selecting Goals, option “slow disease”, I would have liked to see a link for this “and the number of lesions on the first MRI may predict the level of impairment in the long run””	*Add a page in the introduction to clarify source of information provided. Identify sections where additional source links can be added.*
Functionality	“I didn’t like how it asked if I had taken a medication but had no option to say that I had adverse reaction to that medication. Later, it recommended that medication to me.”	*Improve past medication history question – separate previous and current medication, and add question to clarify reason for stopping the medication.*
Value Elicitation Method	“I think it would have been easier to think about the importance of goals based on a number scale rather than a slider that didn’t have a reference point.” (2 patients) “[T]he sliders were a bit confusing. If those were my concerns it is likely I won’t want to lower the value of how I feel about what I want and don’t want. I felt the sliders were not needed.” (4 patients) “I think [this] section could be expanded so there are some different options. It didn’t seem to capture individual differences as much as I would have liked.”	*Refine value elicitation technique and add more explicit instructions.*
	“[I]nstead of having fixed statements maybe have somewhere to type what is important for each person in case their ideas were not covered in the statements to choose from.”	*Add question in “Review” that asks if patient has any other goals they wish their doctor to know that was not addressed in the decision aid.*
	“I have something just not important at all. Just check a box or uncheck a box so it disappears from it.” “Or like a not important, or unsure, don’t know.”	*Include an option to rate some values as “unimportant” or “don’t know”.*
Summary	“There’s no question in any of this that has to do with quality of life. And I wonder if, because somebody could be absolutely having worse time, but they think their quality of life is okay. And somebody, having minor symptoms and they think their quality of life is very poor. And I think that might be an interesting piece of information for the neurologist.”	*Consider including a quality of life instrument.*
	“You’re self-reporting and how accurate are you?… you’re so scared in the beginning and I don’t know how beneficial that is [referring to comparing self-reported disability to population average]. I don’t know what other people think about that.” “It is a little scary/overwhelming.”	*Remove comparison to other MS patients.*

## Discussion

This study used an iterative approach that engaged all relevant stakeholders to develop and assess a PtDA for RRMS. We utilized a mixed-methods approach and found that the PtDA was both highly usable and acceptable. Despite high initial usability and acceptability ratings, patient feedback was used to further refine the RRMS-PtDA. Of note, participants perceived the information presented in the PtDA to be unbiased and credible, two characteristics which are known to be critical for creating effective PtDAs [60]. In addition to high usability and acceptability ratings, treatment-naïve patients reported high levels of preparedness to make a decision after completing the PtDA.

Importantly, participants reported that the PtDA addressed a pressing need for consistent, accessible information in a single location. Furthermore, several focus group participants reported that the PtDA would have decreased the extent to which they felt overwhelmed in initial neurological consultations. With an increasing number of DMT options becoming available, the PtDA helps overcome information overload by ordering options such that those that best match patient’s preferences are shown first [61]. Notably, this does not prevent patients from reviewing all treatment options, but nudges patients towards spending their cognitive effort on the DMTs that are the most likely candidates.

The RRMS-PtDA was designed to facilitate shared decision-making (SDM) between patients and physicians; however, not all patients want to share that decision, and instead some prefer to have the physician make treatment decisions [62]. The Control Preferences Scale, which assesses patient preferences for SDM, is embedded in the RRMS-PtDA, and patient SDM preferences are included in the summary page. By asking about SDM preferences, the RRMS-PtDA ensures that the process is always collaborative as even those who prefer to defer entirely to the treating physician can state their preference and this preference will guide future neurological consultations.

The results of the present study are bolstered by a mixed-methods approach, as well as the engagements of diverse participants with varying disease durations and patient reported disability. The strengths of this study should be considered in the context of its limitations. First, because the PDMS was not administered before and after completion of the PtDA, it is unclear if high PDMS scores are a result of the PtDA itself or simply a characteristic of study participants. Future studies should include pre- post- measures to determine the impact of the PtDA on preparedness. Additionally, the PtDA was developed for first-line treatment of RRMS with DMTs. Consequently, the results from the prototype testing may not apply to individuals who are already receiving a DMT, considering second line therapies or experiencing progressive MS. Although the PtDA utilized a highly credible information source [34], this may not reflect individual physicians’ interpretation of the same data or take into account their clinical experience with different therapies. The goal of a PtDA is to enhance the patient-physician encounter by providing basic information to help frame physician recommendations and enable more consultation time to be spent addressing the patient’s concerns and questions rather than explaining treatment options. Moreover, the PtDA was created specifically for patients at the UBC MS Clinic and may not be generalizable to other patient groups. The PtDA would need to be tailored to each clinic/region, where different treatments may be available, different clinical pathways in place, and patients may have different preferences for attributes of treatment in MS. We also deliberately sought a heterogenous group of participants so that we would obtain a diverse range of perspectives to identify usability issues. For the survey, we had limited ability to recruit just a narrower group and thought that including a group that was not on treatment for at least 2 years would be useful. Lastly, although the PtDA was highly rated, the present study did not assess feasibility.

## Conclusions

We have developed an RRMS-PtDA for first-line DMTs. The PtDA lays the foundation for and promotes SDM by both assessing patient preferences for SDM and by providing clear, credible information on treatments. Thus, the PtDA allows physician consultations to focus on patient priorities, preferences, and questions rather than information provision. The PtDA, available at www.msdecisionaid.com by contacting the research investigator, can be adapted for other jurisdictions and languages, and updated as new treatments and evidence emerges. Future studies will determine how best to implement the PtDA in routine practice, and the effect the PtDA has on treatment initiation and adherence.

## Additional file

Additional file 1: **Table S1.** Participants’ ratings on the System Usability Scale (*N* = 18). **Table S2.** Participants’ ratings on acceptability and usability. **Table S3.** Participants’ ratings on the clarity of the information presented in each section. **Table S4.** Participants’ ratings on the Preparation for Decision Making Scale (*N* = 18). (DOCX 76 kb)

## Data Availability

The datasets used and/or analysed during the current study are available from the corresponding author on reasonable request.

## Abbreviations

CADTH: Canadian agency for drugs and technologies in health
DMT: Disease modifying therapy
IPDAS: International patient decision aid standards
MS: Multiple sclerosis
PtDA: Patient decision aid
RRMS: Relapsing-remitting multiple sclerosis
RRMS-PtDA: Relapsing-remitting multiple sclerosis patient decision aid
SDM: Shared decision-making
UBC: University of British Columbia
