# Supplementary material for: Development and usability testing of a patient decision aid for newly diagnosed relapsing multiple sclerosis patients
Source: BMC Neurol. 2019 Jul 20;19:173. doi: 10.1186/s12883-019-1382-7 (PMC6642472; doi:10.1186/s12883-019-1382-7)
Supplement: Supplementary file 1 — Table S1. Participants’ ratings on the System Usability Scale (N = 18). Table S2. Participants’ ratings on acceptability and usability. Table S3. Participants’ ratings on the clarity of the information presented in each section. Table S4. Participants’ ratings on the Preparation for Decision Making Scale (N = 18). (DOCX 76 kb) [file 12883_2019_1382_MOESM1_ESM.docx]

**Additional File 1 – Supplementary Tables**

Supplementary Table 1. Participants’ ratings on the System Usability Scale (N=18)

| Scale Item | Strongly Disagree | Disagree | Neutral | Agree | Strongly Agree |
| --- | --- | --- | --- | --- | --- |
| I think that I would like to use this decision aid frequently. | 0 | 1 | 3 | 9 | 5 |
| I found the decision aid unnecessarily complex. | 9 | 6 | 3 | 0 | 0 |
| I thought the decision aid was easy to use. | 0 | 0 | 1 | 10 | 7 |
| I think that I would need the support of a technical person to be able to use the decision aid. | 11 | 6 | 1 | 0 | 0 |
| I found the various functions in this decision aid were well integrated. | 0 | 0 | 3 | 10 | 5 |
| I thought there was too much inconsistency in this decision aid. | 7 | 8 | 3 | 0 | 0 |
| I would imagine that most people would learn to use this decision aid very quickly. | 0 | 0 | 1 | 10 | 7 |
| I found the decision aid very cumbersome to use. | 12 | 4 | 1 | 1 | 0 |
| I felt very confident using the decision aid. | 0 | 1 | 4 | 7 | 6 |
| I needed to learn a lot of things before I could get going with this decision aid. | 6 | 4 | 7 | 1 | 0 |

Supplementary Table 2. Participants’ ratings on acceptability and usability

| Items | Frequency  (N=18) |
| --- | --- |
| Amount of information in the decision aid |  |
| Much less than I wanted | 0 |
| A little less than I wanted | 4 |
| About right | 13 |
| A little more than I wanted | 0 |
| Much more than I wanted | 1 |
| Balanced presentation of information in the decision aid |  |
| Clearly slanted towards taking the treatment | 1 |
| Slightly slanted towards taking the treatment | 5 |
| Completely balanced | 12 |
| Slightly slanted towards not taking the treatment | 0 |
| Clearly slanted towards not taking the treatment | 0 |
| PDA fits patients’ discussions with the physician, nurse, or pharmacist |  |
| Yes, as it is | 9 |
| Yes, but with some alteration | 7 |
| No | 2 |
| Icons were readable |  |
| Yes | 18 |
| No | 0 |
| Words in the PDA made sense |  |
| Yes | 18 |
| No | 0 |
| Willing to use PDA or tell someone about it |  |
| Yes | 18 |
| No | 0 |

Supplementary Table 3. Participants’ ratings on the clarity of the information presented in each section

|  | Frequency (N=18) | | | |
| --- | --- | --- | --- | --- |
| Item | Poor | Fair | Good | Very Good |
| What is Multiple Sclerosis? | 0 | 1 | 11 | 6 |
| What are the treatment options? | 0 | 1 | 9 | 8 |
| My preferences | 0 | 3 | 10 | 5 |
| Weighing the benefits and harms of each option | 0 | 2 | 9 | 7 |
| Which option do you prefer? | 0 | 4 | 10 | 4 |
| More information | 0 | 2 | 11 | 5 |
| Information on funding/authors | 1 | 4 | 11 | 2 |
| References | 1 | 3 | 11 | 3 |

Supplementary Table 4. Participants’ ratings on the Preparation for Decision Making Scale (N=18)

| Item | Not at all | A little | Somewhat | Quite a bit | A great deal |
| --- | --- | --- | --- | --- | --- |
| 1. Help you recognize a decision needs to be made? | 0 | 1 | 6 | 6 | 5 |
| 1. Prepare you to make a better decision? | 2 | 0 | 5 | 9 | 2 |
| 1. Help you think about the pros/cons of each option? | 0 | 0 | 2 | 8 | 8 |
| 1. Help patients think about which pros/cons are most important? | 0 | 1 | 4 | 6 | 7 |
| 1. Help you know that the decision depends on what matters most to you? | 0 | 0 | 2 | 8 | 8 |
| 1. Help you organize your own thoughts about the decision? | 0 | 0 | 4 | 7 | 7 |
| 1. Help you think about how involved you want to be in this decision? | 0 | 0 | 1 | 7 | 10 |
| 1. Help you identify questions you want to ask your doctor? | 0 | 1 | 5 | 7 | 5 |
| 1. Prepare you to talk to your doctor about what matters most to you? | 0 | 0 | 4 | 8 | 6 |
| 1. Prepare you for a follow-up visit with your doctor? | 0 | 0 | 6 | 6 | 6 |
